# Gene expression and epigenetic markers of prion diseases

**DOI:** 10.1007/s00441-022-03603-2

**Published:** 2022-03-21

**Authors:** Emmanuelle A. Viré, Simon Mead

**Affiliations:** MRC Prion Unit at UCL, UCL Institute of Prion Diseases, Courtauld Building, 33 Cleveland Street, London, W1W 7FF UK

**Keywords:** Epigenetics, Prion diseases, Genomics, Biomarkers

## Abstract

Epigenetics, meaning the variety of mechanisms underpinning gene regulation and chromatin states, plays a key role in normal development as well as in disease initiation and progression. Epigenetic mechanisms like alteration of DNA methylation, histone modifications, and non-coding RNAs, have been proposed as biomarkers for diagnosis, classification, or monitoring of responsiveness to treatment in many diseases. In prion diseases, the profound associations with human aging, the effects of cell type and differentiation on in vitro susceptibility, and recently identified human risk factors, all implicate causal epigenetic mechanisms. Here, we review the current state of the art of epigenetics in prion diseases and its interaction with genetic determinants. In particular, we will review recent advances made by several groups in the field profiling DNA methylation and microRNA expression in mammalian prion diseases and the potential for these discoveries to be exploited as biomarkers.

**Supplementary information:**

The online version contains supplementary material available at 10.1007/s00441-022-03603-2.

## Introduction: epigenetics

Epigenetics comprises a variety of mechanisms that allow changes in gene expression in the absence of DNA mutation (Bird 2007). Epigenetic regulation of gene expression is a dynamic and reversible process that establishes normal cellular phenotypes and perpetuates heritable traits in dividing cells but also contributes to ageing and diseases (Bettencourt et al. 2020; Zhang et al. 2020). Many definitions of epigenetics have been proposed since the term was first coined by Waddington in 1942 (Bird 2007) and not all necessarily encompass the requirement for maintenance through generations (Gibney and Nolan 2010). Epigenetic mechanisms can be viewed as an interface between the genome and environmental influence. At the molecular level, epigenetic regulation involves covalent modification of DNA (e.g., DNA methylation) and of the histone proteins, chromatin remodelling, and regulation by non-coding RNAs, which we will introduce in more detail in sections below. The dynamic nature of epigenetics, and its reversibility, suggests that it may be possible to use disease-associated epigenetic states to monitor disease progression or alter disease-susceptibility-associated epigenetic states to offer therapeutic interventions; indeed, examples are already available of such applications.

### DNA methylation

DNA methylation has been extensively researched in many biological contexts. Methylation predominantly occurs at the 5’-carbon of cytosine in cytosine-guanine dinucleotides (CpGs) which are unevenly distributed across the genome being concentrated in “islands” near to gene regulatory regions. Methylated cytosine (5mC) is sometimes referred to as the 5th base of DNA (Lister and Ecker 2009). DNA methylation has numerous functions: it is implicated in the repression of transposons and genes, regulating processes such as X-chromosome inactivation, and genomic imprinting. Counterintuitively, more recent work has shown that DNA methylation is associated with actively transcribed gene bodies and, in some cases, with gene activation per se (Rauluseviciute et al. 2020). DNA methylation is therefore of paramount importance for mammalian development (Greenberg and Bourc'his 2019).

There are complex cellular mechanisms to regulate DNA methylation with three different families of proteins: writers (for the establishment and maintenance of DNA methylation), readers, and erasers. In mammals, there are two major “de novo” writer enzymes responsible for the establishment of DNA methylation profiles, DNMT3A and DNMT3B (Okano et al. 1998, 1999), which contain a highly conserved DNMT domain (the MTase domain) in the carboxy terminus and two chromatin reading domains (Greenberg and Bourc'his 2019). To maintain DNA methylation profiles through cell division, symmetrical CpG methylation is required upon DNA replication. This is achieved by the methylation maintenance enzyme DNMT1. UHRF1, an E3 ubiquitin-protein ligase, binds hemimethylated CpG dinucleotides at replication forks and recruits DNMT1 through its ubiquitin-like (UBL) domain. DNMT1, DNMT3A, and DMNT3B use S-adenosyl-l-methionine (SAM) as their methyl-donor (Lister and Ecker 2009).

Addition and/or removal of methyl groups on to the DNA molecules have consequences at the chromatin level. DNA methylation itself can block the binding of proteins and transcription factors locally leading to silencing of gene expression. Yet, recent work shows that CpG methylation of motifs may in some cases increase the binding affinity of some transcription factors, such as CEBPβ (Zhu et al. 2016). Another way DNA methylation contributes to local alterations to chromatin structure is through the recruitment of chromatin modellers: DNMTs themselves can interact with histone modifiers. Protein recruitment can also occur through 5mC and the readers, methyl-CpG-binding domain (MBD) proteins (Hendrich and Bird 1998). The most famous MBD proteins are MBD2 and methyl-CpG-binding protein 2 (MeCP2). All MBDs interact with nucleosome remodelling and histone deacetylase complexes, which leads to gene silencing (Nan et al. 1998).

DNA methylation erasure can either be passive or active. Exclusion of DNMT1 or UHRF1 from the nucleus will impeach maintenance of DNA methylation profiles and lead to a progressive dilution of 5mC across successive generations (Lio and Rao 2019). Conversely, active mechanisms of DNA demethylation involve TET enzymes which oxidise 5mC to 5-hydroxy methyl cytosine (5hmC) which is in turn deaminated to 5-hydroxyuracil (5hU) by activation-induced deaminase (AID). TET enzymes are also capable of successively oxidising 5hmC to 5-formylcytosine and 5-carboxylcytosine (5caC).

Because DNA methylation signals are dynamic and influenced by disease phenotypes, they represent attractive biomarkers. Abnormal methylation can be used for detection and classification of disease, prediction of response to therapeutic interventions, and predicting prognosis. Over the past 2 decades, DNA methylation signals have been used as reliable biomarkers for ageing (Horvath and Raj 2018). The methylation changes at the CpG site in *AHRR* gene are used as biomarker of exposure to smoking and adverse smoking-related health effects even in former smokers (Taryma-Lesniak et al. 2020). Colorectal cancer screening also uses DNA methylation biomarkers with FDA-approved liquid biopsy-base tests targeting methylation changes in genes such as *BMP3* and *NDRG4* (Locke et al. 2019)*.* Similarly, tests for the detection of lung, bladder, cervical, prostate cancers are in development and/or approved by either the European Union (CE approved) or the Food and Drug Administration (FDA) in the USA (Taryma-Lesniak et al. 2020). Finally, because of its undeniable contribution to disease initiation and maintenance, drugs targeting DNA methylation have been developed since the 1980s with some therapies that have progressed to evaluation in clinical trials. DNMT inhibitors are the most effective epigenetic therapy developed to date despite their lack of specificity, poor cellular uptake, and metabolic instability (Michalak et al. 2019).

### Histone modifications

Histones are a family of proteins that bind to DNA in the nucleus and help condense it into chromatin. Together with DNA methylation, histone proteins represent another principle component of chromatin and play a key role in its regulation (Bannister and Kouzarides 2011). Histone modifications play fundamental roles in most biological processes that are involved in the manipulation and expression of DNA. Histone proteins form nucleosomes when in association with compact coils of DNA. They have highly basic amino (N)-terminal tails that protrude from the nucleosome and make contact with adjacent nucleosomes (Bannister and Kouzarides 2011). Modifications of these tails affect internucleosome interactions and thus affect the overall chromatin structure (Bannister and Kouzarides 2011). Moreover, these modifications can also recruit remodelling enzymes or proteins and complexes with specific enzymatic activities which in turn mediate functions of histone modifications. Modifications influence transcription, as well as other DNA processes such as repair, replication, and recombination. The ever growing list of histone modifications has been reviewed extensively elsewhere (Bannister and Kouzarides 2011). As it is the case for DNA methylation, writers, readers, and erasers of histone modifications have been identified and so have their respective specific roles in normal and pathological conditions. As with DNA methylation, histone modifications have been suggested to serve as detection, prognosis, or treatment responsiveness biomarkers in diseases (Chan and Baylin 2012; Chervona and Costa 2012; Kanwal and Gupta 2012; Watanabe et al. 2012). The basis of targeting histone modifiers in diseases lies in manipulating the transcriptional programme to modulate the expression of genes driving disease progression; in essence, reprogramming cancer cells into a more “normal,” differentiated state (Michalak et al. 2019).

### Regulatory RNAs and their modifications

In recent years, work on RNA epigenetic modifications, especially those in non-coding RNAs, has shown that these RNAs play a significant role in regulating gene expression levels. Thanks to developments in RNA sequencing technologies and bioinformatic approaches, the emerging role of non-coding RNAs has become clearer. These RNA molecules are untranslated and capable of regulating gene expression through multiple pathways (Kumar et al. 2020). Non-coding RNAs can target the chromatin, interact with transcription factors, as well as silence gene expression by directly targeting mRNA to degradation (RNA-induced silencing). While the function of most of non-coding RNAs remains poorly characterized, some types of non-coding RNAs have attracted attention, especially in diseases. Particularly, miRNAs and circRNAs are stable in tissues and body fluids and therefore serve as robust biomarkers for numerous diseases (Meng et al. 2017). It is becoming increasingly evident that RNA modifications, or epitranscriptomics, also play a role in development and diseases with more than 100 types of RNA modifications identified, and many modified transcripts are associated with cancers, autism, and other neurodegenerative disorders (Flamand and Meyer 2019). The field of RNA epigenetics is still very much in its infancy, with most of the writers, readers, and erasers, their targets and mechanisms of action to be identified (Esteve-Puig et al. 2020; Berdasco and Esteller 2021).

### Epigenetics in neurodegenerative diseases

In neurodegeneration, it is becoming increasingly evident that epigenetics has its place alongside genetics and environment in the mechanisms involved in disease initiation and progression (Ghosh and Saadat 2021). Changes in DNA methylation are reported to correlate with the onset of AD (Xu et al. 2011; Gjoneska et al. 2015) (Bakulski et al. 2012). The concomitant work of two groups led by Lunnon and Jaeger was the first to convincingly demonstrate that a specific gain in methylation at CpG sites in the *ANK1* gene is associated with AD neuropathology (De Jager et al. 2014; Lunnon et al. 2014). This review however focusses on epigenetic mechanisms in prion diseases.

## Epigenetics in prion diseases (see Fig. 1)

**Fig. 1:**
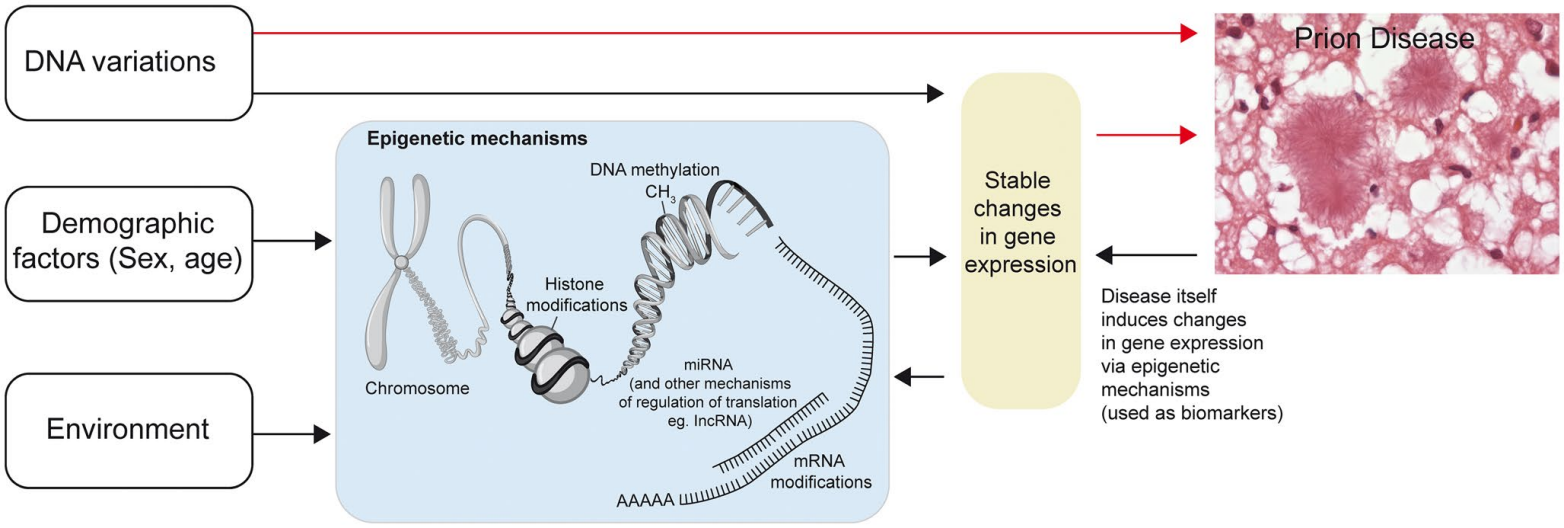
Epigenetics in prion diseases. Epigenetic mechanisms such as DNA methylation, histone modifications, and regulation by non-coding RNAs are influenced by the environment, demographic factors, and disease status. In turn, epigenetics mechanisms alter gene expression profiles in prion diseases. Red arrows show factors altering the risk of disease

Prion diseases are fatal and transmissible neurological conditions, including Creutzfeldt-Jakob disease and inherited prion diseases of humans, and bovine spongiform encephalopathy, sheep scrapie, and chronic wasting disease of cervid species. The infectious agent of the disorders, or prion, is a self-replicating multimeric assembly of misfolded forms of host cellular prion protein (PrP). Recently, the near-atomic structure of a 263-K hamster prion strain has shown amyloid fibrils comprised of parallel in-register intermolecular beta sheets (Kraus et al. 2021). Prion diseases come in distinct clinicopathological subtypes or strains that are thought to be encoded by distinct conformations of the abnormal forms of PrP.

The most common human form of the disease, sporadic Creutzfeldt-Jakob disease (sCJD), presents as a rapidly progressive dementia or neurodegenerative condition associated with cerebellar ataxia, myoclonus, and abnormalities of the motor and sensory systems. There are no treatments that cure or diminish the rate of progression of disease, which is ultimately fatal in all cases, usually within six months. To date, diagnosis of prion diseases is sometimes challenging because there are no non-specific markers of infection in patients, and unlike other pathogens, prions have no associated genome that might allow nucleotide amplification-based diagnostic assays like PCR (polymerase chain reaction). Fortunately, two highly specific forms of investigation are available. Diffusion-weighted MRI (magnetic resonance imaging) brain imaging, now routinely available in most hospitals in the developed world, shows distinct patterns of high signal in the cerebral cortex, striatum, and thalamus in sCJD in over 90% of cases, probably related to the regional distribution of the classical pathological spongiform change (Hermann et al. 2021). A technique named protein misfolding cyclic amplification (PMCA) allows amplification of traces of prions in vitro and their quantification with standard Western blot. To date, PMCA (Soto et al. 2002) could not efficiently amplify prions from sCJD patients (Cali et al. 2019; Belondrade et al. 2020). The real-time quaking-induced conversion assay (RT-QuIC) using CSF as a substrate is also positive in ~ 90% of cases of sCJD and extremely specific (McGuire et al. 2012). This assay employs cycles of shaking and incubation of a test sample in the presence of an excess of recombinant PrP, which is progressively converted to abnormal forms by templating and fission. Once a patient is suspected to have a prion disorder, accurate tests to confirm the diagnosis are available. Further improvements might be made to the recognition of imaging abnormalities at the initial scan reading (Carswell et al. 2012), the sensitivity of RT-QuIC (Hermann et al. 2021), and our ability to diagnose strains of prion disease during life. Diagnostic strategies for animal prion diseases are quite different, depending on the purpose and context: management of wild animals and food safety, in living and slaughtered animals.

We see two main purposes to study epigenetics in prion diseases. First, because early (presymptomatic), specific changes in gene expression are a feature of the laboratory forms of the disease (Hwang et al. 2009), which may be initiated and maintained by epigenetic mechanisms. Expression of PrP is essential for the prion propagation and disease-related toxicity (Bueler et al. 1993; Brandner et al. 1996; Mallucci et al. 2003). While it should be assumed that the vast majority of non-PrP gene expression changes in prion disease are not causal, rather consequences of disease, recent genetic studies suggest that some gene expression changes could play a role in susceptibility, and others may play a role in mechanisms of toxicity or shed light on changes in cell proportions or activation states. A recent genome-wide association study of sporadic CJD implicated genetic factors that determine increased expression of *STX6* in specific brain regions as a risk factor (Jones et al. 2020; Jones and Mead 2020). Other lines of evidence suggest that, as a generality, epigenetic mechanisms may be relevant to susceptibility. Restating the obvious, the major risk factor for sporadic CJD is advanced age, with the annual age-specific incidence reaching a peak of approximately 5–10 per million at age 70 (Zerr and Hermann 2018; Zerr and Parchi 2018; Zerr et al. 2018), and yet, the condition is extremely rare under the age of 40 and may decline in incidence in the extremes of old age. The aging process is profoundly linked to altered epigenetic mechanisms of gene regulation discussed earlier, and methods to intervene in the hope of stalling aging are the subject of intense scientific scrutiny (Lu et al. 2020). In the laboratory, cell susceptibility to prion infection is dramatically influenced by cell type and differentiation state, which again in a general sense are driven by epigenetic mechanisms (Mahal et al. 2007; Marbiah et al. 2014). We are therefore interested in the part epigenetics plays in maintenance of gene expression profiles relevant to prion disease susceptibility and neurodegeneration.

Second, there are unmet needs for biomarkers. While diagnostic tests for patients in hospital with a strong suspicion of CJD are good, more accessible tests, particularly blood tests for diagnosis are not available, we cannot distinguish between strain or subtypes of disease, and molecular measures of disease progression are only beginning to be discovered. Gene expression profiles themselves might have diagnostic uses but are somewhat impractical to use in a clinical setting whereas more robust markers based on epigenetic mechanisms like DNA methylation might have potential. In the remainder of the article, we explore recent evidence and the potential to exploit epigenetics for these purposes.

### Changes to gene expression landscapes in prion diseases

Expression of the prion protein gene itself is crucial for an animal’s or cell’s ability to propagate prions (Solassol et al. 2003). Therefore, targeting the expression of the prion protein is currently being pursued as a key therapeutic strategy (Mallucci et al. 2002, 2007) (White et al. 2008). Important work from Aguzzi and colleagues exhaustively identified miRNAs regulating PrP^C^ levels in cells (Pease et al. 2018). Antisense oligonucleotide (ASOs) mediated knockdown of *Prnp* expression prolonged the incubation time by two months in RML-infected mice (Nazor Friberg et al. 2012). More recent reports in other models of prion disease showed a meaningful delay of both survival time and disease onset in ASO-treated animals (Raymond et al. 2019; Minikel et al. 2020). An array of approaches for the treatment of prion diseases has been recently reviewed (Zattoni and Legname 2021).

Understanding of the processes regulating the expression of the human prion protein gene (*PRNP*) is an important component of our understanding of prion disorders and human therapeutic approaches; yet, very little is known about the precise mechanisms involved. Undoubtedly, epigenetic mechanisms will prove important in regulating where and when *PRNP* is expressed. *PRNP* apparently has a simple gene structure (Puckett et al. 1991) with the GC-rich promoter region close to the transcription start site containing putative transcriptional factor binding sites, including Sp1, Ap-1, Ap-2, and a CCAAT box (Mahal et al. 2001). Hill’s group reported that expression of transcription factors SP1 and/or MTF-1 significantly increases prion protein levels and up-regulates prion gene expression (Bellingham et al. 2009). DNA methylation has been shown to contribute to the regulation of mouse *Prnp* expression. During the differentiation of P19C6 cells into neuronal cells, decreased DNA methylation coincided with increased expression of *Prnp* (Dalai et al. 2017a, b). Yet*,* DNA demethylation using the DNA methyltransferase inhibitor, RG108, had no impact on *Prnp* expression in N2a cells. Finally, the findings from Dalai and colleagues identified sites for CCCTC-binding factor (CTCF) upstream of *PRNP*’s transcription start site(Dalai et al. 2017a, b). CTCF participates in chromatin organization and remodelling, contributing to the repression or activation of gene transcription (Franco et al. 2014). Taken together, these findings suggest that in-depth understanding of *PRNP* regulation is important.

Infection with prions leads to changes in gene expression profiles (Sorce et al. 2020) . In cells, a microarray analysis performed to evaluate gene expression difference in ScN2a cells revealed a list of protein kinases, phosphatases, and signal transduction molecules upregulated upon prion infection (Greenwood et al. 2005). The most remarkable endeavour to map gene expression changes in prion diseases was conducted by Hwang et al. in 2009*.* In this study, microarrays were used to identify sets of genes reflecting the disease process at multiple time points during disease progression in mice from six different genetic backgrounds infected with two different prion strains (Hwang et al. 2009). Many of the core of 333 differentially expressed genes (DEGs) that appeared central to prion disease replicated findings from earlier studies in laboratory mice. Key biological processes identified included complement activation, lysosomal proteases, cholesterol synthesis/efflux, glycosaminoglycan (GAG) metabolism, and sphingolipid synthesis/degradation, microglial activation, and reactive astrocytic gliosis, with some of these readily detectable before clinical signs appeared.

In animals and humans, work from Legname and colleagues conducted an expression study based on discoveries in the *gyrus frontalis superior* region of cynomolgus macaques inoculated with BSE (Barbisin et al. 2014). They identified a gene signature able to distinguish infected macaques with advanced stage disease from control animals with down-regulation of HBB and HBA2 and up-regulation of *TTR*, *APOC1*, and *SERPINA3* in infected macaques. The authors suggested that, because some of these transcripts are also expressed in blood, this gene signature could be explored as a biomarker. In a follow-up study, the same group analysed frontal cortex samples from prion disease patients, patients with Alzheimer disease and age-matched controls (Vanni et al. 2017). Using real-time-quantitative PCR, they found up-regulation of *SERPINA3* in the brain of all human prion diseases, with only a mild up-regulation in AD. Legname and colleagues also used microarray analysis in whole blood prior to and after the onset of clinical signs in BSE-infected cattle. Using a neat analysis process, the authors identified immune response and metabolism gene signatures to be significantly altered in whole blood of infected animals (Xerxa et al. 2016).

### Alterations to DNA methylation profiles in sCJD

While DNA methylation has become increasingly studied in the context of neurodegenerative disorders, very little is known about DNA methylation profiles in human prion diseases. We recently performed a case–control study to analyse the relationship between DNA methylation and sporadic CJD using 405 peripheral blood samples from patients and controls. Using a genome-wide 450-K Illumina BeadChip array, we identified 283 sites with an absolute change in methylation greater than 10% in sCJD (Dabin et al. 2020). We found that methylation at two probes located in the promoter of *AIM2* decreased with disease progression. *AIM2* is a key component of the inflammasome pathway, a component of the innate immune system that drives the production of the inflammatory cytokine interleukin-1β (IL-1β) in response to microbial and nonmicrobial signals (Schroder et al. 2010). In yeast, AIM2 triggering induces a prion-like polymerization of ASC into filaments that provide platforms for activating inflammatory cytokine production. However, prion pathogenesis does not seem to lead to inflammasome activation in mice (Nuvolone et al. 2015). We also showed that sCJD patients display a concomitant decrease in *FKBP5* DNA methylation and elevated cortisol levels. *FKBP5* binds to glucocorticoid receptors and modulates glucocorticoid sensitivity. Epigenetic regulation of *FKBP5* and its association with patient’s behaviour is well documented: accelerated age-related decreases in *FKBP5* methylation are associated with childhood trauma and depressive phenotypes (Zannas et al. 2016), while increased DNA methylation levels of *FKBP5* have been found in patients suffering from post-traumatic stress disorders (PTSD) and major depressive syndromes (Kang et al. 2020). Such changes are reminiscent of the alterations observed in sCJD. Although additional functional work is needed to clarify the relationship between sporadic CJD, *FKBP5*, and the hypothalamic pituitary adrenal (HPA) axis (where *FKBP5* plays a major role), it could be that the HPA axis provides a link between the pathology in the brain and the periphery.

Future work in independent cohorts of sCJD patients is needed before these methods might be considered for clinical use. Together, these results demonstrate a potential utility of profiling DNA methylation in whole blood from patients with sCJD: these profiles can help discriminate sporadic CJD patients from sex- and age-matched healthy controls and may help predict disease duration.

### Changes in miRNA expression in sCJD

MicroRNAs (miRNA) are small single-stranded non-coding RNA molecules (typically containing about 22 nucleotides) that function as post-transcriptional regulators of gene expression. MiRNA profiles have been studied in the brain and blood from animals and patients with prion diseases. More than 13 years ago, Saba and colleagues used microarrays and PCR to profile miRNA expression changes in the brains of mice infected with mouse-adapted scrapie (Saba et al. 2008). They identified 15 miRNAs that were dysregulated during the disease processes and deployed computational analysis to predict potential mRNA targets of these miRNAs. A year later, Montag et al. analysed the differential expression of miRNAs in the brains of BSE-infected cynomolgus macaques. They identified significant upregulation of hsa-miR-342-3p and hsa-miR-494 in the brains of BSE-infected macaques compared to non-infected animals and that hsa-miR-342-3p was also upregulated in brain samples of human type 1 and type 2 sCJD (Montag et al. 2009). The Booth group later reported over-expression of miR-146a in prion-infected mouse brain tissues. The authors performed extensive functional studies and proposed a role for miR-146a as a potent modulator of microglial function by regulating the activation state during prion-induced neurodegeneration (Saba et al. 2012). The same miRNA was then reported by Lukiw et al. to have a major role in the brain’s innate immune response and antiviral immunity and showed that upregulation of miRNA-146a in human prion-based neurodegenerative disorders, including sCJD and Gerstmann-Straussler-Scheinker syndrome (GSS) (Lukiw et al. 2011). The Booth group also reported a decrease in expression of miR-132-3p, miR-124a-3p, miR-16-5p, miR-26a-5p, miR-29a-3p, and miR-140-5p pre-clinically in hippocampal CA1 neurons during infection (Majer et al. 2012).

In 2017, Rubio et al. compared the expression of eight candidate miRNAs (let-7b-5p, let-7d-5p, miR-128-3p, miR-132-3p, miR-146a-5p, miR-21-5p, miR-342-3p, and miR-342-5p) in blood plasma samples from healthy and classical scrapie sheep (Sanz Rubio et al. 2017). miR-342-3p and miR-21-5p were found elevated in scrapie cases which demonstrated the potential feasibility of miRNAs as circulating TSE biomarkers (Sanz Rubio et al. 2017). In 2019, elegant work from Slota et al. identified 47 altered miRNAs in the serum of elk that tested positive for chronic wasting disease (CWD) (Slota et al. 2019). The authors demonstrated that the abundance of these miRNAs could be used to discriminate CWD status in elk. Functional analysis of the 21 miRNAs that were not affected by haemolysis revealed numerous Kyoto Encyclopaedia of Genes and Genomes (KEGG) pathways that were enriched in their putative targets, 3 out of the 21 miRNA signature (miR-148a-3p, miR-186-5p, miR-30e-3p) directly targeting the *PRNP* gene.

In peripheral blood from patients with sCJD, we identified, validated, and replicated three miRNAs (hsa-let-7i-5p, hsa-miR-16-5p, and hsa-miR-93-5p) whose expression is significantly lower in the blood of sCJD patients than in healthy individuals. We also reported that the levels of these three miRNAs distinguish patients with sCJD from patients with AD (Norsworthy et al. 2020). We identified the mRNA targets of these dysregulated miRNAs and compared their expression between sCJD and control blood samples. *CCND3*, *VEGFA*, *NAP1L1*, *ZFP36*, *CDKN1A*, and *RNF44* were selected using TarBase with stringent criteria (validated using luciferase assays and/or validated by immunoprecipitation; expressed in whole blood). Higher levels of four of these transcripts were found in sCJD in comparison to blood from controls, which is consistent with a reduction of miRNA silencing of target mRNA. In mice, Cheng and colleagues demonstrated dynamic expression changes during disease progression in the affected thalamus region and serum (Cheng et al. 2021). The authors validated these differentially expressed miRNAs in extracellular vesicles from human blood samples from patients with sCJD and controls and found that a diagnostic model using miRNA biomarkers associated with prion infection (predicting sCJD with an AUC of 0.800, 85% sensitivity, and 66.7% specificity).

hsa-miR-16-5p was found to be altered in both studies and also features prominently in existing literature on miRNA and neurodegeneration. Studies in prion-infected mice have shown a distinct temporal pattern of dysregulation of hsa-miR-16-5p as disease progresses, whereby expression is upregulated in cultured primary neurons in the pre-symptomatic phase but downregulated in the clinically established disease phase (Norsworthy et al. 2020). Recently, Slota et al. reported decreased miR-16-5p abundance in serum from scrapie-infected hamsters (Slota et al. 2019). Our observations of downregulation of hsa-miR-16-5p in blood from patients with clinically established sCJD concur with these findings. In humans, Llorens and colleagues reported increased levels of hsa-miR-16-5p in sCJD frontal cortex (Llorens et al. 2018). In ALS, hsa-miR-16-5p was found to be downregulated in whole blood (Liguori et al. 2018), and in AD, several clinical and mechanistic studies have suggested that hsa-miR-16-5p has a role in pathogenesis (Kim et al. 2020). Whether altered abundance in the blood is related to the mechanism by which it is differentially expressed in neurons or in neurodegeneration remains to be investigated.

hsa-let-7i-5p has also been implicated in neurodegenerative disorders. In ALS, Liguori et al. reported downregulation of hsa-let-7i-5p in whole blood (Liguori et al. 2018). Conversely, in AD, elevated levels of hsa-let-7i-5p were found in the brain, plasma, and CSF (Gamez-Valero et al. 2019). These findings are consistent with our observations of hsa-let-7i-5p’s downregulation in sCJD blood but upregulation in AD whole blood. More relevant is the report that levels of hsa-let-7i-5p were found to be elevated in exosomes from neurons of prion-infected mice (Bellingham et al. 2012), and in sera from elk with chronic wasting disease (Slota et al. 2019).

## Future directions and conclusions

We now have a good understanding of overall gene expression changes in the brains of animals affected at different stages of prion diseases; however, epigenetic mechanisms that underpin these changes and how they might be exploited to further our understanding and diagnosis of prion diseases remain unclear.

Once sporadic CJD is suspected in humans, excellent diagnostic tests are available, but we are still unable to diagnose using readily accessible biofluids like blood and characterise sporadic CJD strains in life, and biomarkers of prion-disease related neurodegeneration remain in their infancy. Blood DNA methylation, whole-blood, or extracellular vesicle miRNA profiles do show some potential as biomarkers, but the challenges for the future are for independent replication, larger samples sizes, and an assessment of practicality and feasibility (i.e., speed, sample handling, costs) for use in clinical settings.

In terms of the role epigenetics might play in understanding causal mechanisms and the pathogenesis of prion disease, we await with great interest the development of strategies to manipulate *PRNP* expression in patients and those at-risk of prion diseases. In terms of non-*PRNP* mechanisms and co-factors, there are major gaps in our knowledge; for example, the extent to which disease-related gene expression changes and related epigenetic mechanisms are driven by alterations in cell proportions and cell-specific activations states. The burgeoning field of single-cell (Scheckel et al. 2020) analysis of brain tissue and the promise that this might be combined with prion-specific assays have potential to address this issue.

## Supplementary information

Below is the link to the electronic supplementary material.

Supplementary file1 (PDF 19978 KB)
