# Supplementary figures and images for: Gene expression and epigenetic markers of prion diseases
Source: Cell Tissue Res. 2022 Mar 21;392(1):285–94. doi: 10.1007/s00441-022-03603-2 (PMC10113299; doi:10.1007/s00441-022-03603-2)

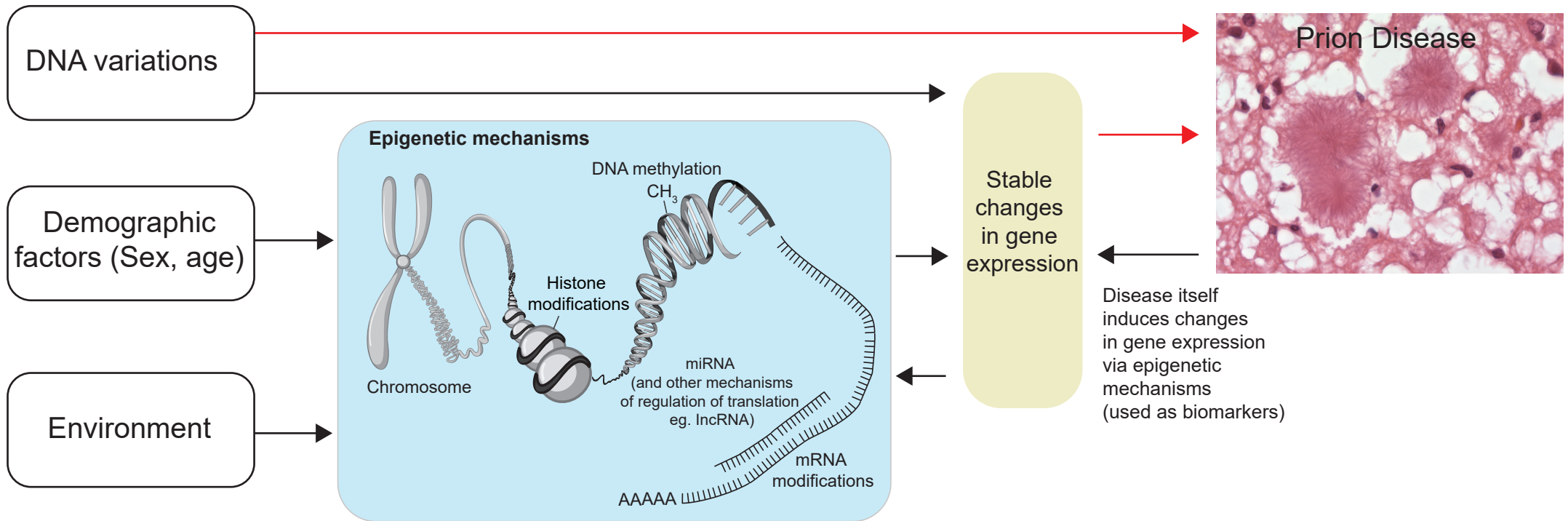

Supplement: Supplementary file 1 — Supplementary file1 (PDF 19978 KB) [file 441_2022_3603_MOESM1_ESM.pdf]
